# Dynamic Conduction and Repolarisation Changes in Early Arrhythmogenic Right Ventricular Cardiomyopathy versus Benign Outflow Tract Ectopy Demonstrated by High Density Mapping & Paced Surface ECG Analysis

**DOI:** 10.1371/journal.pone.0099125

**Published:** 2014-07-11

**Authors:** Malcolm C. Finlay, Akbar K. Ahmed, Alan Sugrue, Justine Bhar-Amato, Giovanni Quarta, Antonis Pantazis, Edward J. Ciaccio, Petros Syrris, Srijita Sen-Chowdhry, Ron Ben-Simon, Anthony W. Chow, Martin D. Lowe, Oliver R. Segal, William J. McKenna, Pier D. Lambiase

**Affiliations:** 1 Institute of Cardiovascular Science, University College London, London, United Kingdom; 2 Department of Medicine, Columbia University, New York, New York, United States of America; University of Oxford, United Kingdom

## Abstract

**Aims:**

The concealed phase of arrhythmogenic right ventricular cardiomyopathy (ARVC) may initially manifest electrophysiologically. No studies have examined dynamic conduction/repolarization kinetics to distinguish benign right ventricular outflow tract ectopy (RVOT ectopy) from ARVC's early phase. We investigated dynamic endocardial electrophysiological changes that differentiate early ARVC disease expression from RVOT ectopy.

**Methods:**

22 ARVC (12 definite based upon family history and mutation carrier status, 10 probable) patients without right ventricular structural anomalies underwent high-density non-contact mapping of the right ventricle. These were compared to data from 14 RVOT ectopy and 12 patients with supraventricular tachycardias and normal hearts. Endocardial & surface ECG conduction and repolarization parameters were assessed during a standard S_1_-S_2_ restitution protocol.

**Results:**

Definite ARVC without RV structural disease could not be clearly distinguished from RVOT ectopy during sinus rhythm or during steady state pacing. Delay in Activation Times at coupling intervals just above the ventricular effective refractory period (VERP) increased in definite ARVC (43±20 ms) more than RVOT ectopy patients (36±14 ms, p = 0.03) or Normals (25±16 ms, p = 0.008) and a progressive separation of the repolarisation time curves between groups existed. Repolarization time increases in the RVOT were also greatest in ARVC (definite ARVC: 18±20 ms; RVOT ectopy: 5±14, Normal: 1±18, p<0.05). Surface ECG correlates of these intracardiac measurements demonstrated an increase of greater than 48 ms in stimulus to surface ECG J-point pre-ERP versus steady state, with an 88% specificity and 68% sensitivity in distinguishing definite ARVC from the other groups. This technique could not distinguish patients with genetic predisposition to ARVC only (probable ARVC) from controls.

**Conclusions:**

Significant changes in dynamic conduction and repolarization are apparent in early ARVC before detectable RV structural abnormalities, and were present to a lesser degree in probable ARVC patients. Investigation of dynamic electrophysiological parameters may be useful to identify concealed ARVC in patients without disease pedigrees by using endocardial electrogram or paced ECG parameters.

## Introduction

Arrhythmogenic Right Ventricular Cardiomyopathy (ARVC) is a disease of cardiomyocyte adhesion; initial myocyte slippage and loss of gap junction integrity is followed by more overt structural changes, with characteristic fibrofatty replacement of cardiomyocytes[1]. These early manifestations of ARVC pose an important diagnostic challenge, especially in differentiating benign outflow tract ventricular ectopy from ARVC, as sudden death may occur in the concealed phase before structural changes appear and an early manifestation may simply be right ventricular (RV) ectopy[2], [3].

Pre-clinical work has highlighted putative mechanisms by which arrhythmias in ARVC may occur. Genetically modified mice deficient in plakophilin-2 exhibit slow conduction in the absence of significant histological anomalies and heterozygous desmoplakin knock out mice have impaired conduction reserve [4]–[6]. The association of the cardiac sodium channel with the desmosome as a functional unit has been proposed as a mechanism by which these electrophysiological changes may arise without conventional histological structural changes [7]. Furthermore, abnormalities in myocyte lineage determination may arise from interactions between mutant transcripts in ARVC and cell fate pathways [8]. Thus several elegant pathogenic mechanisms have been proposed that can explain early arrhythmias in ARVC without overt structural disease.

To date, clinical studies on the electrophysiology of patients with ARVC have overwhelmingly concentrated on patients with established ventricular macroscopic structural markers as a principle feature of their disease. These studies have demonstrated that patients with ARVC exhibit slow conduction within the right ventricle and low endocardial bipolar voltages, indicative of endocardial fibrosis[9], [10]. Whether dynamic conduction slowing is an important distinguishing feature of human disease in the absence of structural anomalies remains to be fully explored.

We have demonstrated significant dynamic conduction and repolarization differences between desmoplakin mutation carriers and controls, but there has been no direct comparison of the dynamic electrophysiological changes in early ARVC versus patients with outflow tract ectopy (RVOT ectopy)[6]. This is an important clinical issue as ARVC patients may initially present with isolated outflow tract ectopy. In this study we specifically investigated a population including patients who qualify as “definite ARVC” in the modified Task Force Criteria *only* because of a definite family history/mutation carrier status *without evidence of significant structural disease* such that they have earlier/milder disease than customarily reported in ARVC. Patients with benign RV outflow tract ectopy (RVOT ectopy) can be viewed as an important control population as they may be expected to exhibit secondary features & adaptations associated with frequent ectopy. Differentiation between ARVC and RVOT ectopy groups will highlight salient disease features rather than merely adaptations to ectopy.

We hypothesized that patients with early ARVC would exhibit slowing of conduction & repolarization changes compared to RVOT ectopy patients under conditions of electrical stress, even prior to detectable structural disease. Furthermore, we hypothesized these myocardial electrophysiological changes would manifest on the surface ECG which ultimately may be of value in the development of a diagnostic test.

## Methods

### Ethics statement

The research was approved by University College London Hospitals Ethics Committee A (08/H0714/97), prior written informed consent to participate in this study was obtained from all participants.

### Patient selection

Patients aged 18–65 years with definite or probable ARVC by modified task force criteria (including familial or genetic criteria) were prospectively recruited to participate in the study and informed consent was obtained. All patients underwent detailed imaging assessment including echocardiography/MRI and were only included in this study if they *did not* have major imaging criteria for ARVC (i.e.: severe RV dilatation, RV aneurysms, severe segmental RV dilatation)[11]. Different aspects of the electrophysiology of the patients who carry desmoplakin mutations have been published elsewhere[6]. Benign right ventricular outflow tract ectopy (RVOT ectopy group) or supraventricular tachycardia patients (Normal group) were compared. The RVOT ectopy group had normal resting and signal averaged ECGs, only unifocal ectopy, structurally normal hearts on echocardiogram/CMR and no family history of sudden cardiac death. All supraventricular tachycardia control patients had normal resting ECGs and normal echocardiograms. In order to minimize the likelihood of a patient with their first presentation of hitherto clinically silent ARVC being misassigned a diagnosis of benign RVOT ectopy, RVOT ectopy patients were excluded if they had a recurrence of ventricular ectopy/tachycardia post ablation, all patients had at least 18 months of follow up.

### Genetic testing

10 ml whole blood samples were obtained from ARVC patients and family members. Genomic DNA was extracted using a commercially available DNA extraction kit (QIAamp DNA Blood mini kit, Qiagen). Index cases were part of a larger patient cohort comprehensively screened for mutations in desmoplakin, plakoglobin, plakophilin-2, desmoglein-2 and desmocollin-2. Primer pairs for DSP exons were designed in flanking intronic sequences and are available on request. Polymerase chain reaction (PCR) amplification and direct sequencing on an ABI 3130 Genetic Analyzer were performed using standard protocols as previously described [12]-[14]. A total of 300 unrelated healthy, ethnically matched Caucasian volunteers served as controls.

### Electrophysiological mapping

The procedure for non-contact mapping has been previously described in detail elsewhere [15]–[17]. In brief, the non-contact array (St Jude Medical, USA) was placed in the right ventricular outflow tract via the left femoral vein under conscious sedation. The non-contact array consists of a basket catheter containing 64 electrodes which is placed within a cardiac chamber. Changes in relative electrode impedance are used to sense the 3D relative positions of a separate roving catheter, and in this way a 3D representation of the chamber endocardial geometry is created. The Ensite system then employs an inverse-Laplacian solution to the field electrograms, sensed by the array and calculates “virtual” unipolar electrograms over the entire endocardial geometrical surface. It thus reconstructs the electrical activity on the wall at any user-designated point. The geometry of the right ventricular endocardium was created with a steerable quadrapolar mapping or ablation catheter. Programmed ventricular stimulation was performed from the right ventricular apex. 3 minutes of steady state pacing at 400 ms coupling intervals was followed by a S_1_S_2_ restitution protocol. This protocol consisted of performing 8 beat trains of pulses (S_1_) at 400 ms coupling intervals followed by a single, premature stimulus (S_2_). The S_1_S_2_ coupling interval was reduced sequentially from 400 ms by 20 ms until 300 ms, then by 5 ms intervals until failure to elicit a ventricular response (refractoriness) was reached. The S_1_S_2_ interval was increased by 8 ms and then further reduced by 2 ms steps to establish the Ventricular Effective Refractory Period (VERP) and proximal end of the restitution curve. Two-second intervals were left between trains. 12 lead electrocardiograms were recorded throughout the procedure.

### Offline analyses

#### Analysis of electrograms

**Figure 1:**
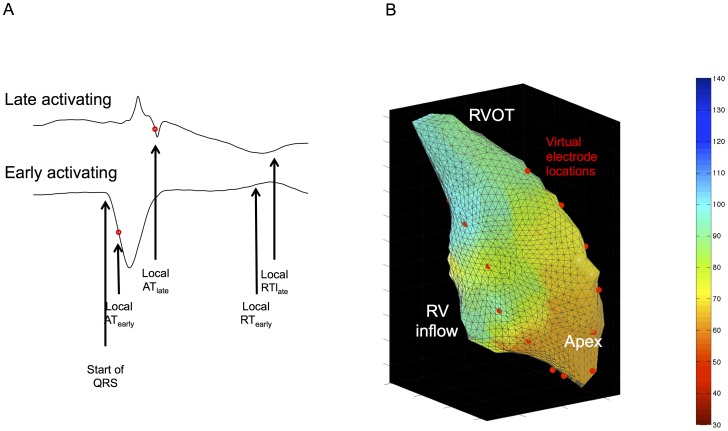
Sinus rhythm electrogram analysis (A) and 3D virtual electrode locations on reconstructed RV shell (B). A) Activation time in sinus rhythm is taken from the earliest discernable ventricular activation (i.e. start of QRS complex) in any lead, and is measured to the local activation from the unipolar electrogram (most negative dV/dt, red circle). Local repolarization is given as the sharpest upstroke of the T wave, as with paced measurements. Time for activation of the RV is thus calculated as the difference between the local AT of the earliest activating electrode (AT_early_) to the local AT of the latest activating electrode (AT_late_). B) Virtual electrodes were placed in four rows corresponding to anterior, lateral, medial and posterior aspects of the RV. They thus covered apex (8 electrodes, 4 segments), outflow tract (8 electrodes, 4 segments) and mid-ventricle (8 electrodes, 8 segments). This enabled global electrograms to be collected without overwhelming quantities of data.

**Figure 2:**
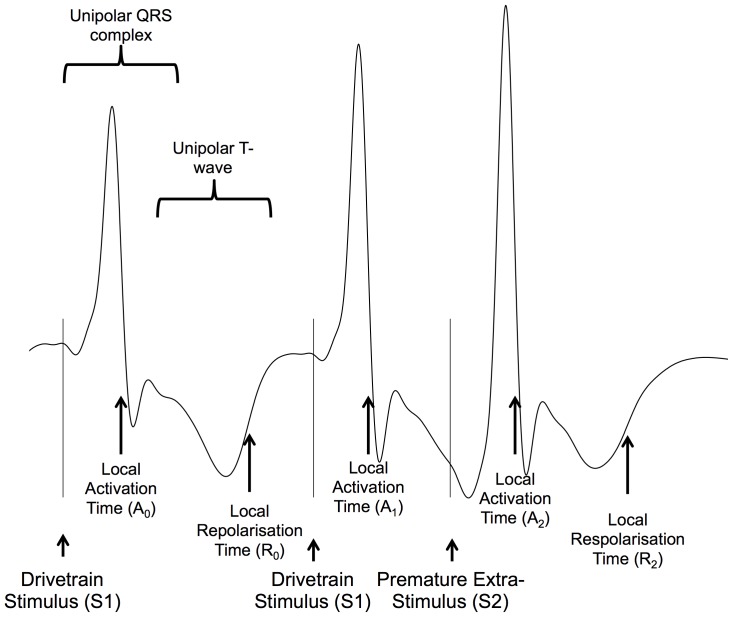
Intracardiac signal analysis. Unipolar electrograms showing the three final activations following a S1S2 train are shown. Timings of stimulus artifact are indicated by S_1_, S_2_. The moment of local activation (A) is taken as the steepest downsloping point of the electrogram complex (A_0_ – A_2_). Repolarisation time (RT) is calculated as the time from stimulus to activation. Local repolarization is taken as the most rapid upstroke of the unipolar T-wave (R_0_ - R_2_) and repolarization time is calculated as the time from stimulus to local repolarization. The ARI is the time between activation and repolarization. Diastolic interval was calculated as A_1_A_2_ – A_0_R_0_ (i.e. local activation interval minus the steady state ARI). AT: Activation Time, RT, Repolarization Time, ARI: Activation Repolarisation Index.

24 “virtual” unipolar electrograms were placed in 4 columns of 6 across the RV chamber on the Ensite console. Global data was thus acquired from the entire geometry (Figure 1) and good spatial resolution achieved without giving overwhelming quantities of data. Electrograms were exported and analysed using semi-automated custom software running in Matlab (The Mathworks Inc., MA, USA). Activation recovery interval (ARI), a well-validated approximation of action potential duration, was defined as the time between AT and repolarization time and measured as previously described (Figure 2) [18]. The slope of ARI restitution was calculated using the least mean squares method [19]. All electrograms, and the results of semi-automated analyses, were manually checked by MF & AA, who were blinded to clinical diagnosis at the time of signal analysis. Reproducibility of electrogram analysis results were confirmed by repeating analyses, with intra-operator variability (Cronbach's alpha) 0.95 for repolarization and 0.96 for activation time measurements.

In sinus rhythm, the time from the earliest electrogram recorded within the right ventricle (RV) to the steepest negative deflection (dV/dt_min_) was used as the local activation time (AT, figure 1). The sinus rhythm AT of the RV was taken as the time from earliest to latest recorded RV activation. During pacing, the time from pacing artifact to dV/dt_min_ was used as the local AT.

Two methods have been used to measure repolarization times during non-contact mapping, termed the classical (Wyatt) method and the alternative method. Both have come under intense theoretical and experimental scrutiny, and here we present results using the classical method [20]–[23]. Though numerical values differed between the two methods, overall results are similar[18]. Activation repolarization interval (ARI), a well-validated approximation of action potential duration, was defined as the time between AT and repolarization time and measured as previously described (figure 2). The slope of ARI restitution was calculated using the least mean squares method. The RV was divided into 16 anatomic segments, activation and repolarization dynamics were studied in the apex, RV body and outflow tract [19].

Fractionation of activation was determined by counting the number of deflections in the differential of the unipolar electrogram [6], [17]. A 30-250Hz filter removed low and high-frequency interference, and a signal-to-noise ratio cutoff of 0.4Hz was applied prior to counting. Analysis was performed in sinus rhythm, steady state pacing and following premature stimulus 2 ms above the ventricular effective refractory period (pre-VERP).

#### Endocardial regional delay and endocardial local activation delay

Mean Increase of Delay is a convenient surrogate measure of conduction velocity restitution. In brief, the activation time is plotted against coupling interval during a restitution curve pacing sequence. Mean Increase in Delay refers to the mean increase in activation time as the coupling interval is reduced to ERP, and is expressed as millisecond increase in delay per millisecond reduction in coupling interval (ms/ms). This was calculated as previously described [6], [17], [24]. Areas of local activation delay during sinus rhythm were determined using activation gradient quantification as previously described [25]. This technique uses an automated algorithm to calculate of the slowest progression of an activation wavefront through the cardiac chamber, and the consistency in the activation wavefront propagation is expressed as a linear determinant r^2^ (the closer the value to 1, the greater the beat to beat consistency).

#### Paced surface electrocardiogram (ECG) analysis

**Figure 3:**
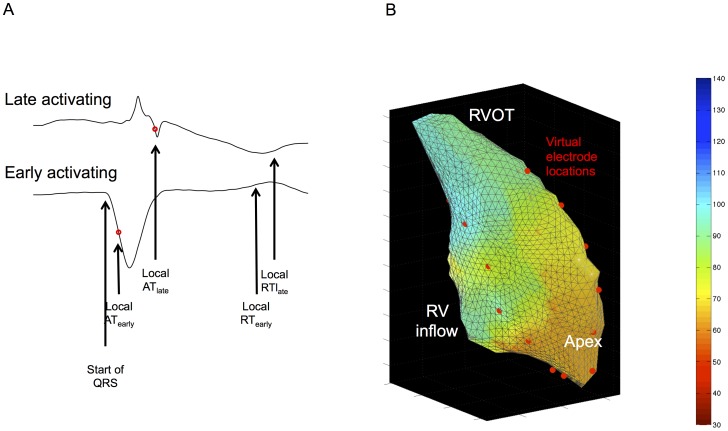
Paced ECG analysis. The final three paced limb-lead ECG complexes following a S1S2 train are shown, with the measurements taken marked. Stim: Stimulus.

12-lead ECGs were recorded simultaneously during the S_1_-S_2_ protocol. Signals were filtered with bandpass settings of 0.1 – 50Hz. Measurements were performed manually using on-screen calipers at 200 mm/sec (MF & AS) blinded to the patient diagnosis. Timings were measured from the pacing stimulus to a) the earliest sharp component of the QRS complex, b) the peak of the QRS complex c) the end of the QRS complex (i.e. the J-point), d) the peak of the T-wave and e) the end of the T-wave (figure 3). These measurements were most consistent throughout the limb leads. Electrical noise was apparent in a high proportion of chest lead recordings during 3D mapping, results are thus presented from limb lead measurements only. Measurements were repeated 3x and the median value used for comparisons. Values presented represent the median value across the patient's limb leads. Intraclass correlation coefficients were 0.98 for intra-observer variability and 0.96 for inter-observer variability of ECG measures. Absolute measurements of surface ECG inscriptions might be very sensitive to the patient-specific placement of the pacing electrode, our analysis focused on measurements of change from steady state pacing following premature stimuli (i.e. hysteresis), guided by the observations of significant changes in intracardiac activation and repolarization.

### Statistical analyses

All statistical computing was performed in R software (R v 2.1.14, R Foundation for Statistical Computing, Vienna [26]). Continuous parametric data are presented as mean±standard deviation or, in the case of non-parametric data, median [interquartile range], unless otherwise specified. Comparisons in which a single measurement was taken for each subject (ventricular effective refractory period, dispersion of repolarization time, activation gradient (r^2^) and mean increase in delay) were made using student's t-test with correction for multiple comparisons. Continuous parametric data derived from electrogram data were modeled using mixed-effects linear regression and statistical significance was inferred from the model[27].

Mixed-effects logistical regression was used to construct predictive models aiming to differentiate patient groups based on electrogram data[28]. Data acquired from electrogram analysis was added to a logistic regression models, resultant receiver-operator curves (ROC) were generated and examined for diagnostic ability. A mixed-effects logistic was created and covariates removed in a stepwise manner to optimise model fit. Mixed-effects modeling allowed individual-patient random factors to be accounted for and included in calculations.

Recursive partitioning (a.k.a. classification and regression trees, CART) was applied to surface ECG data to determine optimum cut-offs attempting to distinguish ARVC from a mixed group of patients. This is a decision-learning technique that enables optimal splits in a group formed on different observations to occur[29]. Once a split is made, the same process in run on the child groups until no further gains can be made (hence recursive). Discovery of optimum decision strategies and cutoffs is rapid, and the outputs are easily understandable and testable. A p value of <0.05 was regarded as statistically significant. The authors had full access to and take full responsibility for the integrity of the data. All authors have read and agree to the manuscript as written.

## Results

### Patient demographics

22 patients with definite (12 pts) or probable (10 pts) ARVC were studied (mean age 46±11 years)[11]. The patients had no major structural features of ARVC with only 2 demonstrating minor modified Task Force imaging criteria. Only 6 patients had T wave inversion in leads V1-V3 and one a slurred S wave on ECG. The signal averaged ECG was positive in 11 cases (5 mutation positive[14], [30], [31]). Twelve definite ARVC patients met the criteria because of their mutation carrier/family history status and therefore represent a group of patients with very early disease preceding the development of diagnostic structural abnormalities. Detailed demographic details are given in Table 1.

**Table 1:** Detailed Demographic factors for ARVC groups.

Subject No	Age (M/F)	Mutation	RV Structural Abnormalities		T-wave inversion V1-V3		Small QRS Complexes		S wave delay		SAECG		NSVT		SVT		PVCs > 500/24 hr		Inducible VT at EP study		FHx		ICD	Criteria			
																								Major			Minor
Definite ARVC																											
1	61 F	T586fsx594 [10]		-	+		-		-		-		-		-		-		-		Minor		+	2			0
2	28 F	T586fsx594[10]		-	-		-		-		-		+		-		-		-		Major		+	2			0
3	53 F	T586fsx594[10]		-	+		-		-		+		-		-		-		-		-		+	2			1
4	37 M	E274fsX288 [18]		-	-		-		-		+		+		-		-		-		Major		-	1			2
5	22 M	S922fsX928 [21]		-	-		-		-		+		+		-		-		+		-		+	1			2
6	40 F	N408K and R941X[18]		-	-		-		+		+		+		-		-		-		Major		-	1			2
7	41 F	PKP2- 2489+1G>A [23]		-	+		-		-		-		-		-		+		-		Major		-	2			1
8	45 M	NIL		Borderline RV dilation	+		-		-		-		+		-		+		-		-		+	1			3
9	42 F	NIL		-	-		-		-		+		+		-		-		-				Major	+1			2
10	57 M	PKP2 S50fsx110 [23]		-	-		-		-		+		-		-		+		-		Major		-	1			2
11	57 M	NIL		Borderline RV dilation	+		-		-		-		+		-		-		-		-		-	1			2
12	64 M	NIL		-	-		-		-		+		-		-		+		-		Major		+	1			2
Probable ARVC																											
13	43 F	E274fsX288 [18]		-	-		+		-		-		+		+		+		-		-		+	1			1
14	50 F	S507F [19]		-	-		+		-		-		+		-		+		-		Major		+	1			1
15	33 M	T586fsx594 [20]		-	-		-		-		-		+		-		+		-		Major		-	1			1
16	52 M	DSG2 T335A [22]		-		-		-		-		-		+		-		-		-		Major	_		1	1	
17	47 F	DSG2 V158G [17]		-	-		-		-		+		-		-		-		-		Major		-	1			1
18	36 M	DSG2 - V392I [17]		-	-		-		-		+		-		-		-		-		Major		-	1			1
19	45 F	NIL		-	-		-		-		+						-		-		Major			1			1
20	55 M	NIL		-	-		-		-		+		-		-		-		-		Major		-	1			1
21	54 F	Nil		-	-		-		-		-		-		-		+		-		-		-	1			1
22	57 F	Nil		-	-		-		-		NA		+		-		+		-		-		-	0			2

SAECG: Signal Averaged ECG, NSVT: Non-sustained ventricular tachycardia, SVT: Supraventricular Tachycardia, PVCs: Premature Ventricular Complexes, FHx: Family Histopy, ICD: Implantable Cardioverter Defibrillator.

25 consecutive patients with monomophic ventricular ectopy (9652±8444 beats/24 h), structural normal hearts and normal resting ECGs underwent non-contact mapping and EP studies. 14 of these patients had >18 m follow-up with no recurrence of ventricular ectopy off antiarrhythmic medication, and no features suggestive of ARVC. These patients were analyzed as the RVOT ectopy group (age 45±14 years). Twelve further patients undergoing electrophysiological studies ± ablation for supraventricular tachycardia with structurally normal hearts acted as Normal controls (age 43±18 years).

### Intracardiac measurements

#### Sinus rhythm: activation and repolarization

No significant differences in RV activation times, ARI or repolarization time were observed between groups (Table 2).

**Table 2:** Endocardial Conduction and Repolarization Parameters.

	Normal	RVOT Ectopy	Definite ARVC	Probable ARVC
	n = 12	n = 14	n = 12	n = 10
**Baseline Heart Rate**	81±17	92±18	85±9	94±11
**VERP**	212±23	210±13	205±16	214±18
**Sinus Activation Time (ms)**	71±19	76±25	81±19	80±19
**Sinus Repolarization Time (ms)**	302±44	311±49	320±40	317±42*
**Sinus ARI (ms)**	230±49	235±44	239±38	237±38
**Steady State AT (ms)**	79±29†	58±24*	75±22†	79±28†
**Steady State ARI (ms)**	194±25	201±20	203±23	204±19
**Steady State Repolarisation Time (ms)**	273±46	260±32	278±35	282±39†
**Mean AT pre-VERP (ms)**	104±33	92±29	123±30††	117±35†
**Mean ARI pre-VERP (ms)**	161±26	166±26	170±28	167±26
**Mean RT pre-VERP (ms)**	265±51	258±44	293±50†	284±52
**Change in AT pre-VERP (ms)**	26±16	34±14	48±21*** †	38±21*
**Change in RT pre-VERP (ms)**	−7±22	−2±22	15±29* †	1±29
**Maximum ARI Restitution Slope (ms)**	0.68±0.41	0.83±0.41	0.77±0.4	0.87±0.48
**Fractionation at pre-VERP**	2.4±0.5	3.4±1.1	3±1* †	3.1±1.1
**Fractionation in Steady State**	2.5±0.6	3.4±0.9	3.1±1.1*	3.3±1*
**Fractionation in Sinus Rhythm**	3±1.4	3.44±1.66	2.84±1.31†	2.91±1.1†
**Mean Increase In Delay**	3.7±10.3	4.1±5.6	6.5±5.6* †	7.4±15.4

Significance codes:

* p<0.05 vs Normals.

** p<0.01 vs Normals.

***p<0.0001 vs Normals.

† p<0.05 vs RVOT Ectopy.

†† p<0.01 vs RVOT Ectopy.

Note: Activation and Repolarization times are given as means±SD for all measurements throughout ventricle.

HR: Heart Rate, ERP: effective refractory period, ARI: Activation Recovery Index, AT: Activation Time, RT: Repolarization Time.

#### Quantification of regional and local conduction delay

Differences in slowest measured activation gradient (a measure of slowest endocardial conduction in sinus rhythm) between groups did not reach statistical significance, although activation gradients in ARVC and RVOT ectopy groups were 20% lower than controls (definite ARVC 0.41±17 mm/ms, probable ARVC 0.41±18 mm/ms, RVOT ectopy 0.40±16 mm/ms, Normals 0.51±13 mm/ms, all p>0.05). r^2^, a measure of uniformity of conduction, was higher in both ARVC groups and in normal controls than in the RVOT ectopy patients (definite ARVC: 0.94±0.07, probable ARVC 0.94±0.05, RVOT ectopy 0.85±0.12, Normals:0.94±0.07, p<0.05 vs RVOT ectopy). There were no differences between either of the ARVC or normal control groups.

#### Steady state pacing: activation and repolarization

In steady state pacing, RV activation took significantly longer in probable ARVC patients than in RVOT ectopy subjects (98±22 ms vs 77±22 ms, p = 0.01), but this did not reach statistical significance in the definite ARVC group (91±21 ms) or normal controls (94±28 ms). There was a trend to longer repolarization times in the ARVC groups when compared to the RVOT ectopy group (definite ARVC: 308±19 p = 0.07, probable ARVC: 307±16 ms p = 0.05, RVOT ectopy: 299±32 ms), but no group was significantly different from normal (303±42 ms).

#### Premature extrastimuli: activation and repolarization

**Figure 4:**
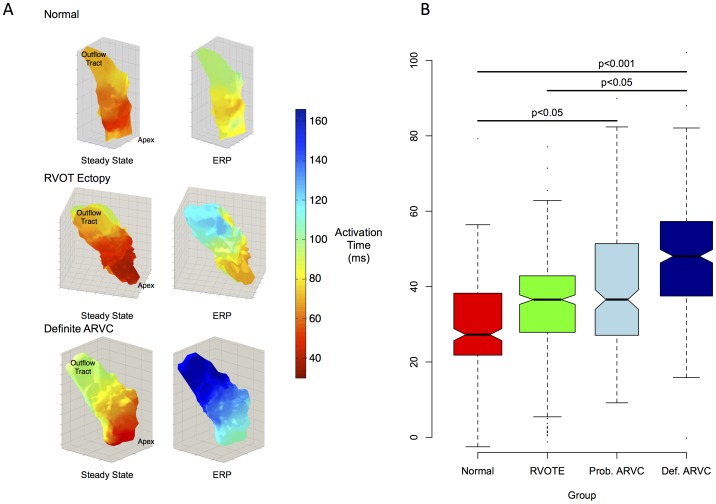
Dynamic changes in activation time. A. Example 3D colormaps showing activation times in steady state and pre-VERP. The change in activation delay in ARVC patients is greater than in Normal Controls & RVOT Ectopy (RVOTE) patients. Color scale represents local activation time relative to pacing stimulus. B. Notched box plot of change in activation times from steady state to ERP. Values are normalized to steady state. Notches indicate approx. 95% confidence intervals.

Examples of restitution curves are illustrated in Figure S1. Ventricular effective refractory periods (VERPs) were similar across groups. Activation times were increased more in the definite ARVC patients at S_1_S_2_ coupling intervals just longer than VERP (pre-VERP) than in other groups (increase in activation time: definite ARVC 48±12 ms, probable ARVC 37±21 ms, RVOT ectopy 34±14 ms, Normal 26±16 ms, p<0.001, Figure 4a). The absolute activation time pre-VERP was also longer in definite ARVC patients than in RVOT ectopy patients (definite ARVC: 123±30 ms, 116±35 ms, RVOT ectopy 92±28 ms, p<0.001 (Figure 4b). There was a trend towards longer absolute activation times in ARVC pre-VERP when compared to Normal controls (104±33 ms, p = 0.1).

The activation delay curves were plotted for each electrode in every patient. Mean increase in delay, a measure of cumulative delay in activation times over all coupling intervals, were greater in the ARVC patients (definite ARVC: Median 7.0 [Interquartile range 3.6–9.3] vs 3.1 [0–5.1] ms^2^, probable ARVC: 6.3 [3.3–9.5] ms^2^) than in RVOT ectopy patients (4.3 [1.4–7.1] ms^2^, p<0.01) or Normal controls (3.4 [0–5.2] ms^2^, p<0.001).

**Figure 5:**
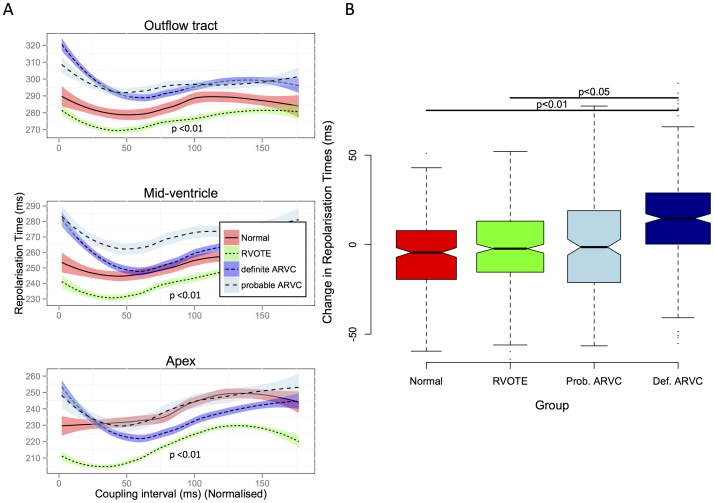
Dynamic changes in repolarization time. A. Loess regression plots of Absolute Repolarisation times within the right ventricle are shown, plotted against coupling interval. There is a marked increase in repolarization times throughout the ventricle at short coupling intervals in the definite ARVC group compared to other groups. B. Change in repolarization time at ERP compared to steady state. There is a significantly greater change in definite ARVC groups than in other groups.

Repolarisation times were compared in a similar manner. The change in repolarisation time pre-VERP compared to steady state was +15±30 ms in the definite ARVC group, whereas it was shorter in other groups (probable ARVC +1±19 ms, RVOT ectopy -2±23 ms p = 0.01, Normal −7±22 ms p<0.01)(Figure 5).

#### ARI restitution slopes

The maximum slopes of ARI restitution curves were marginally steeper in the probable ARVC and RVOT groups compared to normal, but there was no statistically significant difference between the definite ARVC group and any other group (definite ARVC: 0.77±0.40, probable ARVC: 0.87±0.48, RVOT ectopy: 0.83±41, Normal: 0.68±0.41; p<0.05). There were no significant differences in the odds ratio of having a maximum ARI restitution slope of >1 in any segment between patient groups.

#### Fractionated electrograms

**Figure 6:**
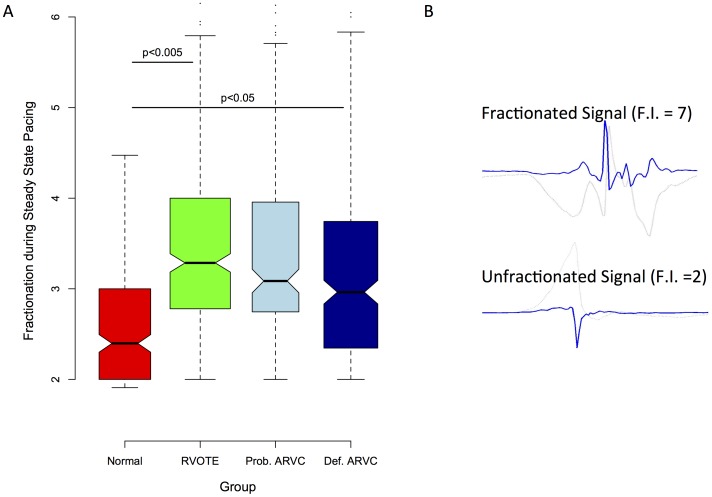
Global fractionation. Panel A shows a notched boxplot of global mean fractionation index. Fractionation is significantly increased in RVOT ectopy and ARVC patients compared to normals. Panel B shows example electrograms (light grey) and their mathematical differentials, from which fractionation index (FI) was calculated. Panel C shows three example colormaps of distribution of fractionation. Higher levels of fractionation are seen in both RVOT ectopy (RVOTE) and ARVC.

**Figure 7:**
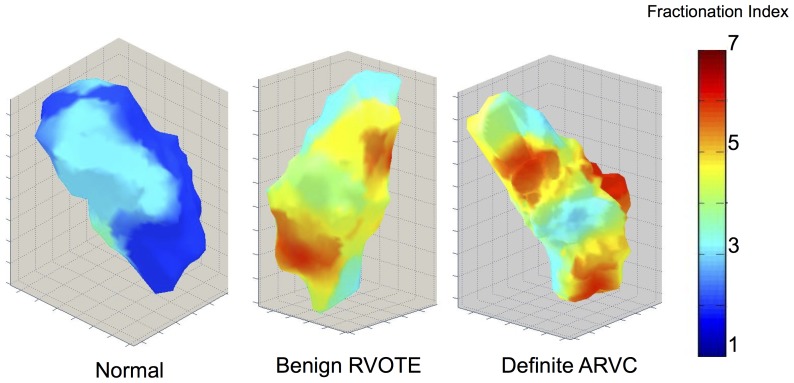
3D color maps of fractionation measured pre-ERP-distribution in Normal individuals, RVOTE and definite ARVC. There is a patchy distribution of fractionated electrograms in both RVOTE and in ARVC ventricles.

In sinus rhythm, more fractionation was observed in RVOT ectopy patients than in definite or probable ARVC or normal controls. However, during pacing at steady state, RVOT ectopy and both ARVC groups had more fractionation than normal controls (Figures 6 & 7). At coupling intervals approaching VERP, the RVOT ectopy group & probable ARVC groups appeared more fractionated than the definite ARVC group (p<0.05), which in turn remained more fractionated than controls (p<0.001).

### Logistic models and the paced-surface ECG

**Figure 8:**
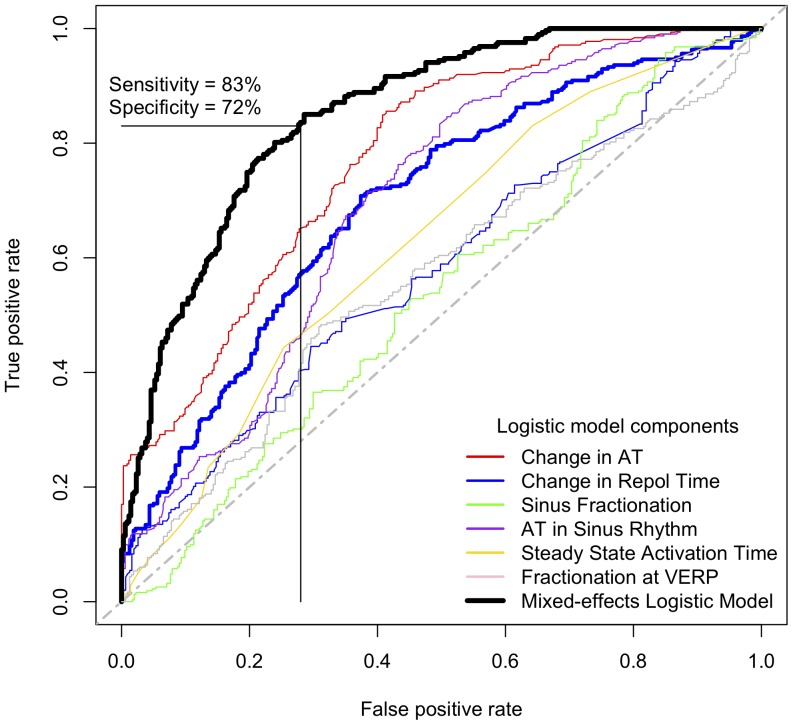
Receiver-operator characteristic (ROC) curves derived from logistic models of electrophysiological criteria. ROC curves are shown for the individual determinants of the most predictive model for definite ARVC derived from electrogram data. AT: Activation time, VERP: Ventricular effective refractory period, Repol Time: Repolarisation Time.

Single intracardiac electrophysiological predictors were generally poor at differentiating definite ARVC from RVOT ectopy, but an increase in the local activation time of >47 ms at ERP compared to in steady state pacing gave a sensitivity of 70% and a specificity of 74% in predicting definite ARVC over RVOT ectopy. A mixed-effects logistic regression model was created to explore the predictive potential of combining intracardiac variables. The resultant model has an area under the curve of 0.85 (Figure 8, Table 3) for differentiating definite ARVC and RVOT ectopy groups indicating a good level of predictive accuracy when changes in activation, repolarisation time and fractionation parameters were included. We considered the intracardiac mapping protocol too complex to recommend as a practical clinical test however, and sought simpler markers that may be of diagnostic value. We therefore examined the surface ECG for discriminators analogous to our intracardiac measurements.

**Table 3:** Mixed Effects Logistic Regression Model coefficients.

	Estimate	Std. Error	z	p
**Random Effects**				
**Location**	0.26±0.51			
**Fixed Effects**				
**(Intercept)**	3.57	0.78	4.57	<0.0001
**Change in AT pre-VERP**	−0.03	0.01	−5.83	<0.0001
**Fractionation in Sinus Rhythm**	0.37	0.06	6.11	<0.0001
**Fractionation at VERP**	0.40	0.08	4.76	<0.0001
**Repolarization time in Sinus Rhythm**	−0.01	0.00	−3.12	<0.001
**Steady State AT**	−0.05	0.00	−10.1	<0.0001

**Figure 9:**
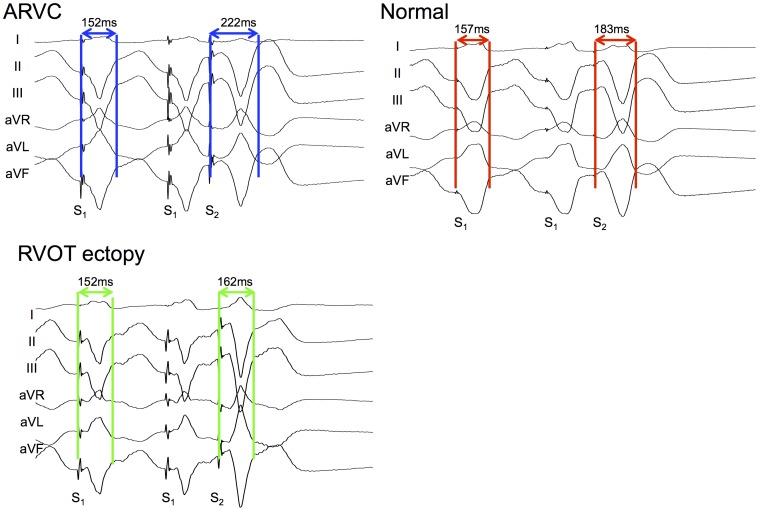
Typical ECG examples of J-point hysteresis. Three representative paced ECGs are shown with stimulus to J-point timings of steady state and pre-VERP beats. The ARVC patient has a markedly extended J-point hysteresis pre-VERP than either normal patient or the RVOT ectopy patient.

**Figure 10:**
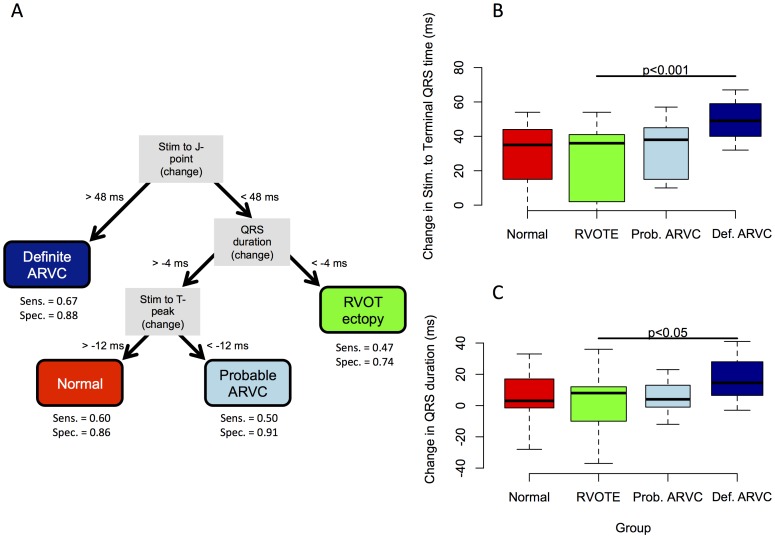
CART analysis . Panel A shows the optimum recursive partition tree. An increase in the time from the stimulus to the end of the paced QRS complex (J-point) of >48 ms gave a sensitivity of 67% and specificity of 88% for definite ARVC. B and C show raw data for the principal branches. These measurements were able to distinguish any ARVC patient from RVOT ectopy (RVOTE)/Normal patients with a sensitivity of 67% and specificity of 84%.

Absolute surface ECG measurements of activation and repolarization did not distinguish between RVOT ectopy and ARVC groups at baseline and pre-VERP. However, significant differences in paced ECG parameters were identified (Figure 9 & 10A, Table 4). A greater *increase* in activation time at ERP by 36±13%compared to steady-state was observed in the definite ARVC group than in the other groups (probable ARVC 21±10%, Normal 21±11%, RVOT ectopy 17±16%, figure 8B), measured as an increase in time from pacing artifact to QRS onset (latency), pacing artifact to nadir of S wave and pacing artifact to end of QRS complex (J-point). No significant change was observed in other groups. Application of the model to the combined probable/definite group gave an AUC of 0.73 (p<0.001) and optimal sensitivity and specificity of 65% and 67%.

**Table 4:** Paced Surface ECG parameters.

		Definite ARVC	Probable ARVC	Normal	RVOT Ectopy
		(mean ± SD, ms)	(mean ± SD, ms)	(mean ± SD, ms)	(mean ± SD, ms)
**Steady State**	**Latency**	34±9	39±11	37±10	42±13
	**Stim. To QRS peak**	81±19	95±15	85±13	90±11
	**Stim. To J-point**	142±32	159±9	147±18	149±22
	**Stim. To Tpeak**	269±24	268±40	269±21	270±24
	**Stim. To Tend**	347±20	361±17	351±28	342±26
	**QRS duration**	107±28	120±12	110±15	107±22
	**Tpeak to Tend**	78±13	94±37*	82±14	72±10
	**QT interval**	313±19	322±24	314±25	300±29
**Pre-ERP**	**Latency**	66±15	66±11	62±14	66±18
	**Stim. To QRS peak**	128±20	135±16†	115±18	128±22
	**Stim. To J-point**	191±36	193±15	171±24	180±29
	**Stim. To Tpeak**	271±25	264±18	264±32	265±26
	**Stim. To Tend**	383±23	375±28	368±30	359±33
	**QRS duration**	125±32	126±14	109±14	115±28
	**Tpeak to Tend**	112±17*	111±23*	104±18	94±19
	**QT interval**	316±24	309±27	306±27	293±32
**Hysteresis (Pre-ERP minus Steady State)**	**Latency**	32±12	27±12	24±9	24±8
	**Stim. To QRS peak**	47±13††	40±13	30±19	38±15
	**Stim. To J-point**	50±12*††	**33±17**	**24±24**	**32±19**
	**Stim. To Tpeak**	**1±19**	**-4±31**	**-5±25**	**-5±15**
	**Stim. To Tend**	35±23†	14±21	17±22	17±22
	**QRS duration**	**17±14††**	**6±11**	**-1±20**	**8±18**
	**Tpeak to Tend**	34±19	17±45	22±16	22±17
	**QT interval**	3±22	-13±20	-7±20	-7±21

Significance codes:

* p<0.05 vs Normals.

** p<0.01 vs Normals.

† p<0.05 vs RVOT Ectopy.

†† p<0.01 vs RVOT Ectopy.

Time from pacing artifact to the end of the T-wave was used as a surrogate marker of complete repolarization of the ventricles. This interval increased in definite ARVC patients more than in either RVOT ectopy patients or the probable ARVC group.

### Diagnostic utility of paced ECG changes (CART analysis) (Figures 7 & 8)

A prolongation in the time from the pacing artifact to the end of the QRS complex of >48 ms (pre-VERP minus steady state) gave a sensitivity of 67% and specificity of 88% in the diagnosis of definite ARVC (figure 10). A reduction in QRS duration of -6 ms or less was insensitive (sensitivity 50%) but specific (specificity 85%) for RVOT ectopy. Further partitioning based on a shortening in the time from stimulus to the peak of the T-wave of greater than - 11 ms distinguished possible ARVC and normal groups, with sensitivity and specificity at distinguishing these two groups from one another of 71% and 59% respectively.

## Discussion

This is the first study to compare differences in conduction and repolarization kinetics in an ARVC population with earlier disease than customarily reported with benign RVOT ectopy cases. The key findings include: 1) Marked conduction delay in patients with early ARVC when pacing at short coupling intervals, which was not evident in sinus rhythm. This exists on a spectrum, with definite ARVC exhibiting more conduction delay than probable ARVC patients compared to normals. 2), Progressively longer local repolarization times as coupling intervals decrease towards VERP in definite ARVC patients versus normal controls & RVOT ectopy subjects. 3) Marked differences in fractionation at short coupling intervals pre-VERP throughout the right ventricle between patient groups, with more fractionation in both ARVC and RVOT ectopy patients. 4) Surface ECG biomarkers of conduction/repolarization dynamics provide diagnostic information - an increase in the time from pacing stimulus to full ventricular activation (defined as the end of the QRS complex i.e. the “J-point”) of greater than 48 ms gave an 88% specificity and 68% sensitivity of distinguishing definite ARVC from other groups.

Previous clinical studies have demonstrated electrophysiological abnormalities in established ARVC with significant structural disease, including prolonged RV activation times in sinus rhythm and low endocardial bipolar electrogram voltages [9], [32]. However, this has not been validated for the clinical differentiation of benign RVOT ectopy or VT from early ARVC. At least 40% fibro-fatty replacement is required before detectable attenuation of endocardial voltages occurs and thus disease expression in the early, concealed phase is missed [32], [33]. Although 12 lead ECG anomalies may precede structural changes, these can be transient or non-specific [34]. The problem of recognising early ARVC is reflected in recent amendments to the consensus diagnostic criteria, which highlight the increasing importance of familial disease and genetic markers so that a definite diagnosis can be made utilizing genetic status or a family history of the disease [11]. This means the diagnosis can be made in the absence of obvious structural disease. Early recognition of disease has potentially important management implications; including avoidance of exercise training, ICD prophylaxis and targeted family screening.

An earlier study of desmoplakin mutation carriers demonstrated that these patients have significantly greater mean increases in delay during an S_1_-S_2_ restitution protocol, particularly in the outflow tract compared to SVT controls[6].The question arose as to whether these changes in conduction and repolarization dynamics are a universal feature of ARVC. This would be consistent with pre-clinical studies implying arrhythmia mechanisms independent of defined scar. If so, these criteria could be utilised to differentiate benign RVOT tachycardia from concealed ARVC. We therefore investigated cases with little or no imaging abnormalities plus patients classed as borderline ARVC using updated consensus criteria.

Our observation of an increase in induced conduction slowing with premature extrastimuli can be regarded as evidence of a lack of conduction reserve. This is consistent with data implying a sodium current deficit in murine models of ARVC [4], [7], [35], and cross-over between Brugada syndrome and ARVC in the mechanisms of arrhythmia [5].

Importantly, it was primarily the family history of ARVC or mutation carrier status that allocated these patients to the “Definite ARVC” group in our study. In the absence of gene testing, 4/12 of the definite ARVC cases would be regarded as borderline. This implies that in a population of combined probable and definite cases the predictive accuracy of this partitioning method may be increased. Indeed, when both groups are combined, the classification tree was able to distinguish any ARVC patient from a combined group of RVOT/Normals with a sensitivity of 67% and specificity of 84%.

We can speculate why a disparity may exist in the degree of induced activation delays between these two groups. Six patients in the definite ARVC group exhibited a surface 12-lead ECG feature of disease at rest, versus none in the probable ARVC group (Table 1). Lack of conduction reserve would both become more apparent during the pacing protocol and unmask features seen on the 12-lead ECG. Both the demographic data and mapping results imply that the electrical phenotype in our definite ARVC patients was more severe than in probable ARVC patients.

Even the patients with more advanced disease did not demonstrate global RV activation delays in sinus rhythm, but it was more marked at short coupling intervals during RV pacing. The dynamics of repolarization were also significantly affected, amplifying effects of conduction delay in ARVC and creating significantly more prolonged repolarization times pre-VERP than steady state versus controls and RVOT ectopy patients. Digital examination of the signal averaged ECG may yet reveal previously hidden features of conduction delay in these patients, but this fell outside of the scope of this study.

The partitioning tests identified stimulus to T-wave peak interval as a potential distinguishing feature between normal and probable ARVC patients. This measure reflects a composite of activation and repolarisation phenomena, and could be seen as a surface ECG marker of the intracardiac repolarisation time changes observed (figure 5).

### Endocardial electrophysiological changes in RVOT ectopy

Two phenomena were observed in the RVOT ectopy patients: consistently shorter repolarisation times versus supraventricular tachycardia patients and ARVC, cases and increased fractionation compared to control cases. The shorter repolarisation times may reflect a memory phenomenon induced by the high ventricular ectopic burden. A shortening in ARI occurs and with it a reduction in refractory period, thus allowing local myocytes to be more susceptible to activation. The high degree of fractionation in benign RVOT ectopy patients similar to that seen in ARVC also deserves comment. Conduction delay and fractionation occurs in ARVC due to fibrofatty replacement and/or dissociation of preferentially conducting myocardial pathways through reduced gap junction coupling and Na channel downregulation [4]-[6], [35], [36]. However, the conduction delays measured in benign RVOT ectopy patients were indistinguishable from normal hearts, yet significantly more fractionation existed. A single plane of fibroblasts could explain this by facilitating ectopic formation through source-sink mismatching & fractionation in one direction of activation, but preserving conduction in another [37], [38]. This hypothesis could be tested using differential pacing in a future study. In the ARVC patients, higher r^2^ values indicating uniformity of conduction were observed compared to RVOT ectopy cases. Similarly, we have reported more uniform conduction in Brugada Syndrome versus controls [17] suggesting that diseases that promote dissociation of the myocardial layers whether due to structural or ion channel differences reduce the contribution of activation from the Purkinje network breaking through the endocardium to allow more homogeneous & consistent endocardial activation & wavefront propagation.

Taken together, features of conduction delay, increased repolarization times and mild fractionation at short coupling intervals pre-VERP would support a diagnosis of early ARVC over that of a benign RVOT ectopy, with a logistic model demonstrating a positive predictive value of 80% from the presented data. These differences can also be identified simply using paced-QRS-T-wave parameters.

This study confirms that reduced conduction reserve develops early in ARVC before detectable structural RV changes on conventional imaging ensue. This has important implications since sudden cardiac death can occur in patients with minimal histological changes in the sub-clinical phase of ARVC and thus a more sophisticated clinical evaluation using dynamic conduction-repolarization changes is required [3]. In a recent study, a positive signal averaged ECG correlated with the size of reduced endocardial voltage regions and histological evidence of cardiomyopathic disease but had a low sensitivity; 21% cases studied still had evidence of major RV structural abnormalities on imaging, indicating that static surface ECG markers may be too insensitive in the concealed phase [39]. In a study examining ventricular ectopic morphology in ARVC, the extent of structural disease was not described [40]. This study illustrates that *dynamic* surface ECG conduction-repolarization parameters may be of diagnostic value in early disease.

### Study limitations

The human mapping technique evaluates endocardial electrophysiology and thus mid-myocardial and epicardial effects of ARVC could not be assessed. This is important since structural changes manifest epicardially prior to progressing endocardially [40]. Although differences in myocardial electrophysiology between benign RVOT ectopy patients followed for >18 months post ablation and ARVC were identified, a prospective long-term follow-up study of this patient cohort would be required to confirm these observations. As with any novel disease marker, validation in a *de novo* population will be required for an impartial assessment of the diagnostic utility of paced J-point hysteresis. Logistical regression models can highlight the differences between groups in this study, but cannot be taken as a diagnostic test based on the data presented.

The probable ARVC group still represents a diagnostic dilemma as a gold standard test is lacking, yet our data has important pathophysiological implications for the understanding of the development of arrhythmias in this population. Disease progression in increasingly understood as initially an epicardial phenomenon, we were not able to examine epicardial effects with our mapping or pacing strategy. Non-contact mapping may be insensitive at identifying low-voltage fractionation in small areas.

## Conclusions

Early ARVC exhibits greater conduction delay and dispersion of repolarization, particularly at short coupling intervals, than either normal hearts or benign RVOT ectopy. Fractionation is mildly increased in early ARVC, but also significantly in benign RVOT ectopy. The dynamic differences in conduction, repolarization and fractionation are manifest on the surface paced ECG & could help refine the early identification of early concealed ARVC patients from those with benign outflow tract ectopy.

## Supporting Information

Figure S1 **Examples of restitution curves from a patient with ARVC and from a normal control.** Repolarisation time, ARI and activation time are plotted against coupling interval. Points represent means of four repeated measurements. Early activated sites are shown in red, late in blue.
(TIFF)
