# Correction: Dynamic Conduction and Repolarisation Changes in Early Arrhythmogenic Right Ventricular Cardiomyopathy versus Benign Outflow Tract Ectopy Demonstrated by High Density Mapping & Paced Surface ECG Analysis

**DOI:** 10.1371/journal.pone.0105457

**Published:** 2014-08-06

**Authors:** The PLOS ONE Staff

Figure 3 is an accidental duplication of Figure 1. Please see the correct Figure 3 here.

**Figure 3:**
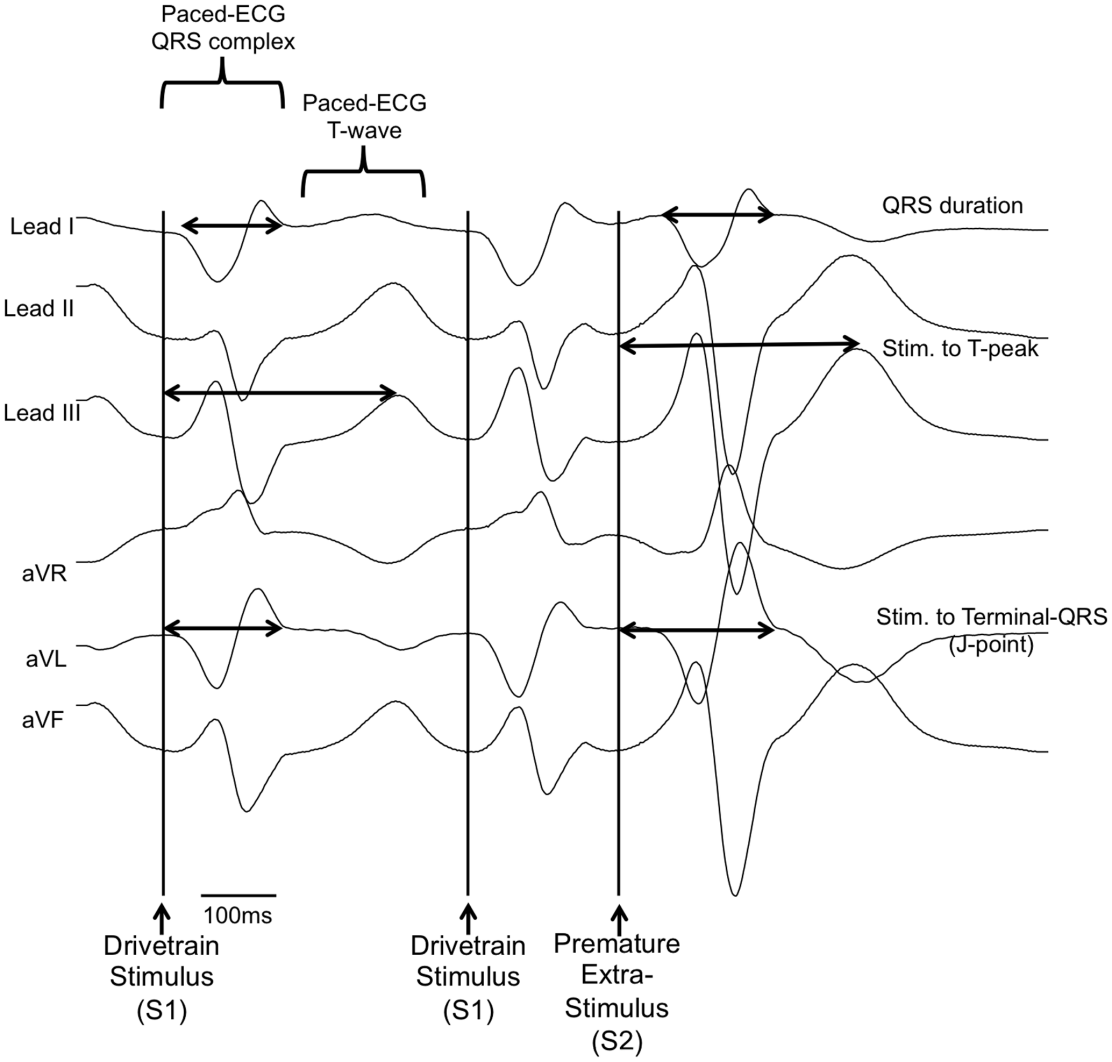
Paced ECG analysis. The final three paced limb-lead ECG complexes following a S1S2 train are shown, with the measurements taken marked. Stim: Stimulus.

## References

[1] Finlay MC, Ahmed AK, Sugrue A, Bhar-Amato J, Quarta G, et al (2014) Dynamic Conduction and Repolarisation Changes in Early Arrhythmogenic Right Ventricular Cardiomyopathy versus Benign Outflow Tract Ectopy Demonstrated by High Density Mapping & Paced Surface ECG Analysis. PLoS ONE 9(7): e99125 doi:10.1371/journal.pone.0099125 25014132 10.1371/journal.pone.0099125 PMC4094482

