# Supplementary figures and images for: Dynamic Conduction and Repolarisation Changes in Early Arrhythmogenic Right Ventricular Cardiomyopathy versus Benign Outflow Tract Ectopy Demonstrated by High Density Mapping & Paced Surface ECG Analysis
Source: PLoS One. 2014 Jul 11;9(7):e99125. doi: 10.1371/journal.pone.0099125 (PMC4094482; doi:10.1371/journal.pone.0099125)

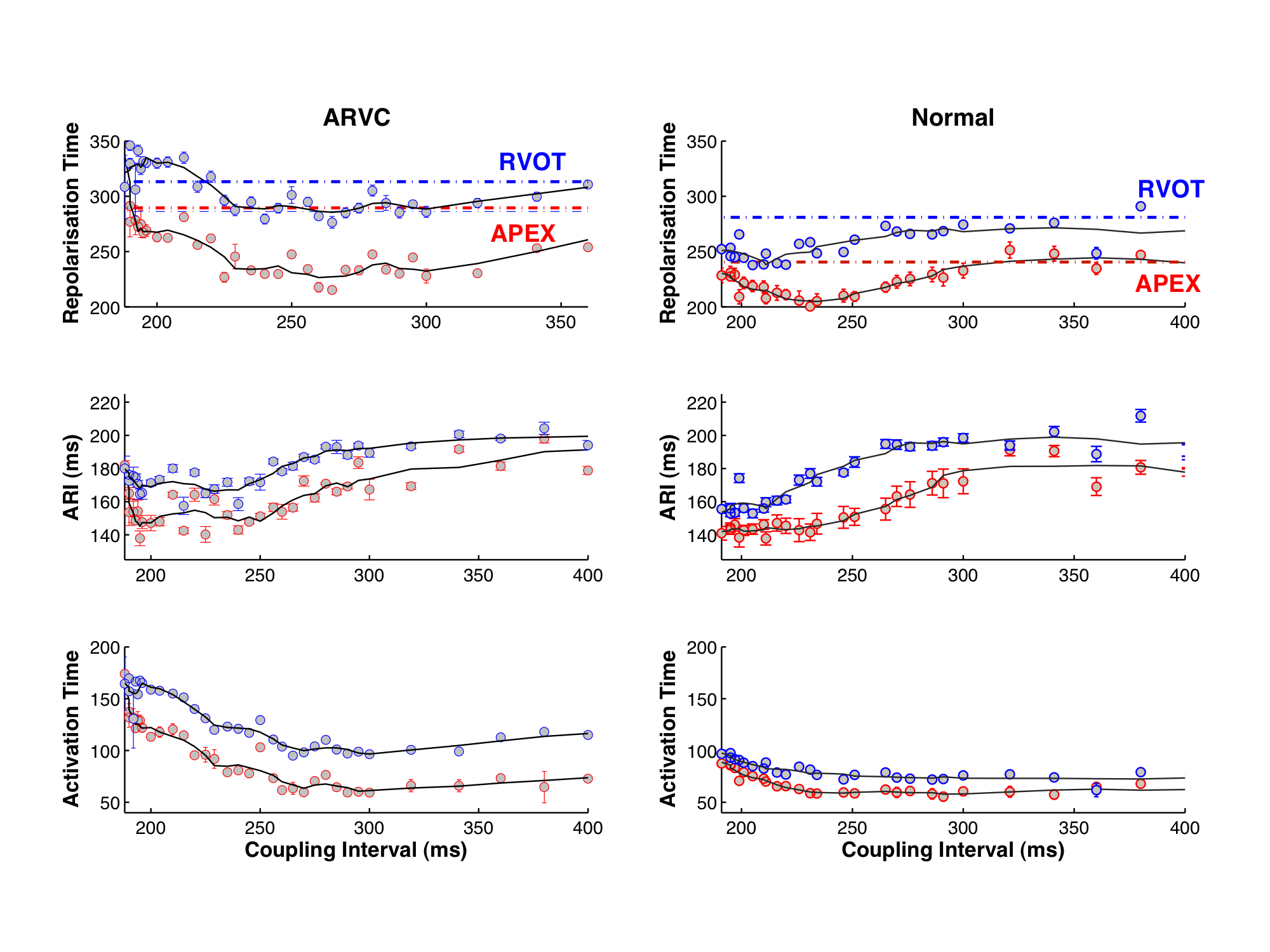

Supplement: Figure S1 — Examples of restitution curves from a patient with ARVC and from a normal control. Repolarisation time, ARI and activation time are plotted against coupling interval. Points represent means of four repeated measurements. Early activated sites are shown in red, late in blue. (TIFF) [file pone.0099125.s001.tiff]
